# Proportion of food insecurity and its sociodemographic correlates among Spanish adolescents: the EHDLA study

**DOI:** 10.3389/fnut.2025.1527685

**Published:** 2025-03-31

**Authors:** Emily Cisneros-Vásquez, Miguel López-Moreno, Héctor Gutiérrez-Espinoza, Jorge Olivares-Arancibia, Rodrigo Yañéz-Sepúlveda, Nerea Martín-Calvo, Lee Smith, José Francisco López-Gil

**Affiliations:** ^1^One Health Research Group, Universidad de Las Américas, Quito, Ecuador; ^2^Diet, Planetary Health and Performance, Faculty of Health Sciences, Universidad Francisco de Vitoria, Pozuelo, Spain; ^3^Faculty of Education, Universidad Autónoma de Chile, Santiago, Chile; ^4^AFySE Group, Research in Physical Activity and School Health, School of Physical Education, Faculty of Education, Universidad de las Américas, Santiago, Chile; ^5^Faculty Education and Social Sciences, Universidad Andres Bello, Viña del Mar, Chile; ^6^Department of Preventive Medicine and Public Health, Faculty of Medicine, Universidad de Navarra, Pamplona, Spain; ^7^IdiSNA, Instituto de Investigación Sanitaria de Navarra, Pamplona, Spain; ^8^Pathophysiology of Obesity and Nutrition, Centro de Investigación Biomédica en Red, Instituto de Salud Carlos III, Madrid, Spain; ^9^Centre for Health Performance and Wellbeing, Anglia Ruskin University, Cambridge, United Kingdom

**Keywords:** food insecurity, hunger, adolescents, Spain, correlates

## Abstract

**Introduction:**

Insufficient and unequal access to food (i.e. food insecurity [FI]) has a negative impact on health, especially in vulnerable groups such as adolescents. This study determined the prevalence of FI and its sociodemographic correlates among adolescents in the *Valle de Ricote*, Region of Murcia, Spain.

**Methods:**

A secondary analysis was conducted using data from the Eating Healthy and Daily Life Activities (EHDLA) study, which provides a sample of 882 adolescents (median = 14, interquartile range [IQR] = 2) collected during the 2021–2022 academic year. FI was measured using the Child Food Security Survey Module in Spanish (CFSSM-S).

**Results:**

Findings showed a prevalence of FI of 16.2%, with significant sociodemographic disparities. Adolescents from immigrant (n: 67; %: 46.9; odds ratio [OR] = 2.41; 95% CI: 1.38 to 4.21) and diverse (n: 25; %: 17.5; OR=2.04; 95% CI: 1.10 to 3.79) families showed a higher susceptibility to FI. In addition, higher parental education was related to lower FI (university education: n: 13; %: 9.6; OR=0.36; 95% CI: 0.17 to 0.74).

**Conclusion:**

These findings highlight the need for targeted public health policies that improve access to nutritious food, enhance parental education, and address socioeconomic inequalities to effectively reduce FI among Spanish adolescents.

## Introduction

Food insecurity (FI) is a global problem that affects millions of people worldwide from both high and low- and middle-income settings (1). FI is defined as the condition in which people face limitations in physical, social and economic access to sufficient, safe and nutritious food that meets their dietary needs and food preferences to maintain an active and healthy life (2). This situation has serious health consequences, especially among vulnerable populations, as it contributes to the increased prevalence of diseases related to malnutrition, such as anemia, undernutrition and overweight (3, 4). The impact of FI is of particular concern in the child and adolescent population, as these groups, during crucial phases of growth and development, may experience nutritional deficiencies, cognitive deficits, poor academic performance, stunted growth and development, overweight and obesity, chronic physical and mental health problems, and even an increased risk of mortality (5, 6).

Globally, more than 2 billion people experience some degree of FI, which is a persistent challenge to achieving the Sustainable Development Goals related to food and nutrition (2). Although FI is more common in developing countries, it also affects developed nations (1). According to a study based on the Global School-based Student Health Survey (GSHS), the prevalence of moderate to severe FI among adolescents worldwide is 44.9 and 6.2%, respectively (7). In Spain, FI has increased (2.6%) due to the economic crisis and job insecurity (8), affecting 13.3% of the general population in the period 2020–2021, as assessed using the Food Insecurity Experience Scale (FIES) (9). Data from the *Instituto Nacional de Estadística* (INE), gathered through the *Encuesta de Condiciones de Vida* (ECV), indicate that 6.9% of the Spanish child and adolescent population experiences poor nutrition (INE (10)). In Catalunya, a cross-sectional study relying on the Child Food Security Survey Module–Short Form (CFSSM-S) reported a prevalence of 18.3% of FI among adolescents aged 12 to 16 years (11). Third-sector organizations, such as *Creu Roja de Catalunya* (12) and *Caritas* (13), used the Latin American and Caribbean Food Security Scale (ELCSA) to underscore the severity of the issue at the municipal level, reporting that 60.6% of families (N = 1001) with children consume an unbalanced diet. Of this group, 29.5% experience mild FI, characterized by concerns about food availability and reduced diet quality, while 40.7% suffer moderate FI, with a decrease in both food quality and quantity (12).

FI is subject to variations depending on sociodemographic factors and individual characteristics such as body mass index (BMI) and lifestyle habits (1, 14–16). Sociodemographic factors are crucial for understanding FI in adolescents. According to the National Institute on Minority Health and Health Disparities (NIMHD), FI is closely linked to poverty, affecting 35.3% of low-income families (17). In Spain, more than 1 in 10 children and adolescents experience severe material deprivation, which limits their access to adequate food (10). Migratory status is also a key factor, as Spanish adolescents who are children of immigrants are twice as likely to suffer FI compared to those from native families, due to language barriers and discrimination (18). In addition, the educational level of the parents has a significant influence: the prevalence of FI in Spain is 23.8% in households where the parents have a low educational level, compared with 2.1% in those with university-educated parents (19). Adolescents living in rural areas have higher probabilities of experiencing FI compared with those residing in urban areas (2), and those living in cohabiting families are 78% more likely to experience FI compared with those living in nuclear families (20). These data underscore the need for a multifactorial approach to address FI in adolescents.

FI represents a significant public health challenge, especially among Spanish adolescents, where its prevalence and associated factors are critical but insufficiently explored areas of research. Although general data on FI at the global level and some estimates at the national level in Spain are available (2, 9, 21), there is a notable lack of specific studies that comprehensively address this problem in the Spanish adolescent population. This research gap underlines the urgent need for studies that not only quantify the prevalence of FI, but also identify the sociodemographic variables associated with FI. Therefore, the primary aims of the present study were to determine the prevalence of FI in a sample of Spanish adolescents and to identify the sociodemographic factors related to this status. It is expected that findings from this study will guide policies and programs aimed at mitigating FI, thus promoting equitable and sustainable access to adequate and healthy food in the Spanish context.

## Materials and methods

### Study design and population

This study conducts a secondary analysis using data obtained from the Eating Healthy and Daily Life Activities (EHDLA) study, which covers a representative sample of adolescents in *Valle de Ricote* (Region of Murcia, Spain). Data collection was conducted during the 2021–2022 academic year in the three secondary schools in the region. The methodological approach employed in the EHDLA study has been described in detail in previous publications (22). In the initial phase, 1378 adolescents were selected through simple random sampling. From this group, 496 participants (36.0%) were excluded due to missing data on FI and and all the correlates examined. As a result, the final sample consisted of 882 adolescents, who were included in the analyses presented in this study.

The inclusion criteria required participants to be between 12 and 17 years old and registered and/or residing in *Valle de Ricote*. The exclusion criteria were as follows: (1) exemption from the Physical Education subject, as assessments and questionnaire completion were conducted during these classes; (2) the presence of medical conditions contraindicating physical activity or necessitating special care; (3) undergoing pharmacological treatment; (4) lack of parental or legal guardian consent to participate in the research project; and (5) refusal to voluntarily participate in the study.

### Procedures

#### Food insecurity

The measurement of household FI was conducted using the Child Food Security Survey Module validated in Spanish (CFSSM-S) (6). This questionnaire was developed to assess individuals’ perceptions of FI, considering aspects such as the risk of food supply depletion, the need to resort to low-cost food, difficulty in sustaining a nutritionally balanced diet, decrease in food intake, reduction in food ration size, hunger, skipping meals, and food deprivation for a twenty-four-hour period. The CFSSM-S instrument contains nine items, rated on a three-point Likert scale. Each affirmative response, indicating moderate or high FI (“sometimes” or “a lot”), adds one point. Food security levels were determined following the criteria of Connell et al. (23) and the United States Department of Agriculture (24). Households were classified into three categories: “food security” with a score of 0 to 1 point, “low food security” with a score of 2 to 5 points, and “very low food security” with a score of 6 to 9 points. However, due to the small number of households classified as having “very low food security,” it was decided to recode the original three categories into two: “food security” and “food insecurity.”

#### Sociodemographic variables

General data were collected on the sex and age of the participants. Schooling was classified into two types: public and private. Area of residence was distinguished between urban (more than 5000 inhabitants) and rural (5000 inhabitants or less) (25). Students were classified as immigrants if they met at least one of the following conditions: A child of immigrant parents, born outside Spain or having at least one parent born abroad. Socioeconomic status (SES) was assessed using the Family Affluence Scale (FAS-III) (26). The FAS-III score was measured by summing the responses to 6 questions: (a) Ownership of a vehicle (0 = no, 1 = yes, one, 2 = yes, two or more), (b) Availability of own room (0 = no, 1 = yes), (c) Number of computers in the household (0 = none, 1 = one, 2 = two, 3 = more than two), (d) Number of bathrooms (with shower and/or bathtub) in the dwelling (0 = none, 1 = one, 2 = two, 3 = more than two), (e) Availability of a dishwasher in the home (0 = no, 1 = yes), (f) Frequency of trips outside Spain in the last year (0 = none, 1 = once, 2 = twice, 3 = more than twice). The final score ranged from 0 to 13 points, resulting in the creation of three distinct categories based on SES: low (0–2 points), medium (3–5 points), and high (≥6 points). Furthermore, adolescents were individually questioned about the educational level of their father/mother/legal guardian. The available options were incomplete primary education (<6 years), complete primary education (6 years), incomplete secondary education (<4 years), complete secondary education (4 years), incomplete higher education (<2 years of high school), or complete higher education (2 complete years of Baccalaureate or complete university).

To assess the family environment, adolescents were asked about various household characteristics. Information was collected on the number of people residing in their households and the number of siblings. In addition, they were asked to indicate the type of family, with the following options: nuclear (i.e., two parents [a father and a mother] and their biological children), single parent (a single parent [i.e., father or mother] assumes the upbringing of their children), extended [includes not only parents and their children, but also other relatives living in the same household (grandparents, aunts, uncles, nieces, nephews, cousins)] and diverse [family structures that do not conform to traditional models (same-sex parent, reconstituted, foster, adoptive and compound families)].

The selection of these factors was based on prior literature and the conceptual model of social determinants of health (27), which establishes that FI is influenced by a combination of individual, family, and contextual factors (24). Moreover, these variables are directly related to the sociodemographic characteristics of the study population and were included to minimize bias and improve the interpretability of the results.

### Statistical analysis

To evaluate the normality of the variables, visual techniques such as density and quantile-quantile plots were used, complemented by the Shapiro–Wilk test. The median with its interquartile range (IQR) was presented for quantitative variables, and frequencies (*n*) and percentages (%) were described for qualitative variables. For comparisons between groups, the chi-square test was employed for categorical variables. When FI status was collapsed into two categories (i.e., “food security,” or “FI”), the Mann–Whitney *U* test was used for non-normally distributed quantitative variables. Conversely, when the original three FI categories were preserved (i.e., “food security,” “low food security,” or “very low food security”), the Kruskal-Wallis *H* test was applied.

A robust generalized linear model (GLM) with binomial distribution was employed to examine the sociodemographic factors linked to FI status. The model’s overall significance was assessed using the Pearson chi-square test (*χ*^2^). Additionally, the model’s goodness-of-fit was assessed using Nagelkerke’s *R*^2^, which provides an adjusted measure of explained variance for logistic regression models. The Akaike Information Criterion (AIC) and the Bayesian Information Criterion (BIC) were also employed to assess model performance and facilitate comparisons with alternative models.

Odds ratios (ORs) derived from the logistic regression models were interpreted in terms of their effect size following the approach proposed by Chen et al. (28). Specifically, an OR of 1.68 corresponds to a small effect size (Cohen’s *d* ≈ 0.2), an OR of 3.47 indicates a medium effect size (Cohen’s *d* ≈ 0.5), and an OR of 6.71 represents a large effect size (Cohen’s *d* ≈ 0.8). OR values below 1.5 were considered negligible (Cohen’s *d* ≈ 0.2), while values exceeding 5 were associated with very large effect sizes (Cohen’s *d* ≈ 0.8).

All statistical analyses were performed using R statistical software (version 4.3.2) developed by the R Core team in Vienna, Austria, together with RStudio (version 2023.12.1 + 402) from Posit in Boston, Massachusetts, U.S.A. A *p* value <0.05 was considered significant for all statistical analyses.

## Results

The descriptive analysis of the participants, according to their food security status, is presented in Table 1. Among the 882 participants, 16.2% (*n* = 143) were categorized as having FI. The median age distribution was homogeneous between both groups (14 years, *p* = 0.901). The distribution by sex in the sample was balanced, with 44.1% of boys and 55.9% of girls, with no significant differences between the groups analyzed (*p* = 0.655). Most of the participants attended public schools and resided in urban areas. No significant differences were found among groups in terms of type of schooling (*p* = 1.000) or area of residence (*p* = 0.234). However, significant differences were observed in several socioeconomic and demographic indicators. Food insecure participants had a higher representation of low SES (37.1% vs. 16.6%, *p* < 0.001) and were more frequently immigrants (46.9% vs. 18.9%, *p* < 0.001). In addition, the prevalence of FI was higher among non-Caucasian participants (32.2% vs. 12.7%, *p* < 0.001) and those whose parents had lower levels of education, both for the mother (41.5% vs. 27.9%, *p* < 0.001) and father (50.7% vs. 32.6%, *p* < 0.001). Food insecure households also had a higher number of siblings (median 2.0 vs. 1.0, *p* < 0.001). There was a significant difference in the number of people living in the household (*p* = 0.018), although the median was 3.0 in both groups. Likewise, family type showed that adolescents from non-nuclear families had a higher propensity to FI (28.7% vs. 14.3%, *p* < 0.001). The characteristics of the study participants based on the original categories of the CFSSM-S (i.e., “very low food security,” “low food security,” or “food security”) can be found in Supplementary Table S1.

**Table 1:** Descriptive data of adolescents in the *Valle de Ricote*, Region of Murcia, Spain, participating in the study of food insecurity (FI) prevalence and its sociodemographic correlates according to FI status.

Variable		Food security	Food insecurity	Total	*p*-value^†^
Participants	*n* (%)	739 (83.8)	143 (16.2)	882 (100.0)	
Age	Median (IQR)	14.0 (2.0)	14.0 (2.0)	14.0 (2.0)	0.901
Sex	Boys	323 (43.7)	66 (46.2)	389 (44.1)	0.655
	Girls	416 (56.3)	77 (53.8)	493 (55.9)	
SES status	Low SES	123 (16.6)	53 (37.1)	176 (20.0)	<0.001
	Medium SES	405 (54.8)	67 (46.9)	472 (53.5)	
	High SES	211 (28.6)	23 (16.1)	234 (26.5)	
Immigrant status	Native	599 (81.1)	76 (53.1)	675 (76.5)	<0.001
	Immigrant	140 (18.9)	67 (46.9)	207 (23.5)	
Type of schooling	Public	586 (79.3)	113 (79.0)	699 (79.3)	1.000
	Private with public funds	183 (20.7)	30 (21.0)	183 (20.7)	
Area of residence	Urban	535 (72.4)	111 (77.6)	646 (73.2)	0.234
	Rural	204 (27.6)	32 (22.4)	236 (26.8)	
Number of siblings	Median (IQR)	1.0 (1.0)	2.0 (1.0)	1.4 (1.0)	<0.001
Number of people at home	Median (IQR)	3.0 (1.0)	3.0 (1.0)	3.3 (1.0)	0.018
Race/ethnicity	Caucasian	645 (87.3)	97 (67.8)	742 (84.1)	<0.001
	Non-Caucasian	94 (12.7)	46 (32.2)	140 (15.9)	
Mother’s educational level	Primary education or lower	205 (27.9)	59 (41.5)	264 (30.1)	<0.001
	Secondary education	301 (41.0)	59 (41.5)	360 (41.1)	
	University education	228 (31.1)	24 (16.9)	252 (28.8)	
Father’s educational level	Primary education or lower	233 (32.6)	69 (50.7)	302 (35.5)	<0.001
	Secondary education	306 (42.8)	54 (39.7)	360 (42.3)	
	University education	176 (24.6)	13 (9.6)	189 (22.2)	
Type of family	Nuclear	633 (85.7)	102 (71.3)	735 (83.3)	<0.001
	Single-parent	35 (4.7)	10 (7.0)	45 (5.1)	
	Extended	19 (2.6)	6 (4.2)	25 (2.8)	
	Diverse	52 (7.0)	25 (17.5)	77 (8.7)	
CFSSM-S (score)	Median (IQR)	0.0 (0.0)	3.0 (2.0)	0.0 (1.0)	<0.001

CFSSM-S, Child Food Security Survey Module in Spanish; IQR, interquartile range; SES, socioeconomic status. ^†^Statistical significance determined by Pearson chi-square (*χ*^2^) test (for categorical variables) or Mann–Whitney *U* test (for continuous variables).

Table 2 presents the results of the generalized linear model that identifies the sociodemographic correlates of FI. The results revealed that immigrant status (OR = 2.41, 95% confidence interval (CI): 1.38 to 4.21) and family structure (OR = 2.04, 95% CI: 1.10 to 3.79) are significant predictors of FI among adolescents. Based on the guidelines proposed, these odds ratios can be interpreted as medium effect sizes (OR = 2.04, Cohen’s *d* ≈ 0.4; OR = 2.41, Cohen’s *d* ≈ 0.5). In addition, the educational level of the father was also significantly associated, with a reduced probability of FI as the educational level increased (university education: OR = 0.36, 95% CI: 0.17 to 0.74). This OR corresponds to a large effect size (Cohen’s *d* > 0.8). Although other factors such as number of siblings, area of residence and maternal education showed trends in the associations, they did not reach statistical significance. The regression model was significant overall (*χ*^2^ = 87.447, *p* < 0.001) and showed a reasonable fit with a Nagelkerke’s *R*^2^ of 0.168, indicating that the included factors explain approximately 16.8% of the variance in FI. Additionally, model performance was assessed using the AIC and the BIC, with values of AIC = 690.931 and BIC = 766.239.

**Table 2:** Generalized linear model for sociodemographic correlates of food insecurity among adolescents in the *Valle de Ricote*, Region of Murcia, Spain.

Predictor	OR	95% LLCI	95% ULCI	*p*-value
Age (per one year)	0.28	0.85	1.10	0.630
Sex				
Boys	Reference			
Girls	0.84	0.56	1.25	0.389
SES status				
Low SES	Reference			
Medium SES	0.76	0.47	1.24	0.274
High SES	0.58	0.31	1.10	0.093
Inmigrant status				
Native	Reference			
Immigrant	**2.41**	**1.38**	**4.21**	**0.002**
Type of schooling				
Public	Reference			
Private with public funds	1.00	0.60	1.66	1.000
Area of residence				
Urban	Reference			
Rural	0.80	0.50	1.27	0.339
Number of siblings (per one sibling)	1.16	0.93	1.46	0.182
Number of people at home (per one person)	1.06	0.86	1.32	0.581
Race/ethnicity				
Caucasian	Reference			
Non-Caucasian	1.29	0.70	2.39	0.414
Mother’s educational level				
Primary education or lower	Reference			
Secondary education	1.06	0.66	1.69	0.821
University education	0.79	0.42	1.50	0.469
Father’s educational level				
Primary education or lower	Reference			
Secondary education	0.65	0.41	1.03	0.063
University education	**0.36**	**0.17**	**0.74**	**0.005**
Type of family				
Nuclear	Reference			
Single-parent	1.95	0.79	4.81	0.149
Extended	1.48	0.51	4.28	0.473
Diverse	**2.04**	**1.10**	**3.79**	**0.024**

LLCI, lower limit confidence interval; OR, odds ratio; SES, socioeconomic status; ULCI, upper limit confidence interval. Bold indicates a *p*-value < 0.05. Model performance: Pearson chi-square (χ^2^) = 87.447 (*p* < 0.001); Akaike information criterion (AIC) = 690.931; Bayesian information criterion (BIC) = 766.239; Nagelkerke’s *R*^2^ = 0.168.

## Discussion

The present study provides significant evidence on the proportion of FI in adolescents in the *Valle de Ricote*, Region of Murcia, Spain, identifying that 16.2% of the participants are affected by this condition. The findings show that FI transcends the mere lack of food resources, being configured as a multidimensional phenomenon influenced by various sociodemographic factors, among which the educational level of the father, immigrant status and family structure stand out. These results are particularly relevant in the field of public health, since they show the existence of structural barriers that hinder access to adequate food for certain vulnerable groups. The identification of these predictors constitutes a solid empirical basis for the design of public policies and preventive programs aimed at reducing social inequalities, promoting more inclusive environments that favor healthy and equitable development in the adolescent population.

Building on this, our results indicate that the proportion of FI (16.2%) in this specific region may be shaped by unique social, political, and cultural factors inherent to the *Valle de Ricote*. The area’s agricultural economy and the seasonality of employment are likely contributors to economic instability in households, increasing their vulnerability to FI (29). When situating these results within a broader context, notable variations in FI prevalence emerge across countries. For instance, higher rates have been reported in Canada (30) and Mexico (31), likely driven by greater poverty and inequality levels. Conversely, a study in Germany found a prevalence of 27.8%, surpassing that of the present study, which may reflect differences in social policies and food support systems (32). The INE (2024) (10) reports a national prevalence of FI of 6.9% among the Spanish child and adolescent population. The higher prevalence found in our study (16.2%) in adolescents from the *Valle de Ricote*, Region of Murcia, may be due to differences in the methodologies used. While the INE utilized the ECV Survey to assess FI, our study employed the validated CFSSM-S scale. Furthermore, it is important to note that national-level studies may not capture regional disparities, as in the case of our study, which focused on a specific local population. In Catalunya, a proportion of FI of 18.3% was found among adolescents (11), similar to the present finding of 16.2%, whereas other national studies reported slightly higher rates (18). These variations may be due to methodological differences in data collection and operational definitions of FI (33), affecting the comparability of results.

In addition, certain sociodemographic factors significantly linked to this condition were identified. First, being an immigrant stood out as a significant predictor, as immigrant adolescents were more than twice as likely (OR = 2.41) to face FI compared to their native counterparts. This finding is aligned with the study by Barreiro-Alvarez et al. (18) (OR = 1.92), conducted in adolescents from Terrassa, Spain, highlighting the vulnerability of immigrant families in terms of food security. Research indicates that immigrant families face economic challenges, such as low-wage jobs, job insecurity and the need to send remittances, which deplete their financial resources. Moreover, they have limited access to food aid programs due to lack of information and legal barriers (34). Loss of access to culturally familiar and nutritious foods, coupled with an unfavorable socioeconomic environment and lack of knowledge about the nutritional value of foods available in the host country, could contribute significantly to FI (35). Acculturation experiences and culturally restricted food preferences also limit access to a balanced diet, especially in European contexts where cultural diversity influences eating habits, and immigrants have difficulty finding and affording foods in line with their traditional practices (35). These economic, social, and cultural barriers create a difficult environment for immigrant families, increasing their vulnerability to FI.

The educational level of the father is a determining factor in household food security. Our study shows that fathers with university education have lower rates of FI in their children (OR = 0.36). This observation is consistent with research conducted in Mexico (31), Canada (30) and Germany (36), which highlight a higher probability of FI in households with low educational level. Some studies suggest that families with parents who have completed high school or incomplete postsecondary education have an elevated likelihoood of FI compared to those with lower or college education. This could indicate that families with less formal education develop adaptive skills that enable them to cope better with FI (37). In addition, the literature suggests that college-educated parents have access to better job opportunities and higher incomes, allowing them to acquire a greater variety of nutritious foods and to manage household resources more efficiently (38–40). The education of the head of household also acts as a protective factor, facilitating integration and access to resources in the country of residence, as well as improving knowledge of and access to food assistance programs (41, 42). On the other hand, parents with lower levels of education tend to provide less healthy foods to their children (43), while those with higher levels of education are better able to distinguish and choose healthy foods (44), thus contributing to family food security.

Another sociodemographic factor associated with the proportion of FI in adolescents is family type. Our study indicates that belonging to a diverse family doubles the odds of experiencing FI (OR = 2.04). This finding is consistent with previous research, which indicates that nontraditional family structures experience greater difficulties in providing adequate nutrition (31, 32, 45, 46). However, studies in other contexts, such as Nigeria, have found that polygamous households, a diverse family type, have better food security outcomes compared with monogamous households (47). Although polygamy is not common in Spain due to cultural and religious factors (48), this contrast underscores the importance of considering the cultural and socioeconomic context when analyzing the relationship between household type and food security. Economic and social stability is essential to ensure food security, and diverse families often deal with greater challenges in these areas. Reconstituted families, for example, may experience economic and emotional strains due to the integration of members of different nuclear families. Studies indicate that children in households with cohabiting parents are 78% more likely to experience FI than those in married families (20). In addition, the economic investment of stepparents in non-biological children tends to be lower, which can lead to an unequal distribution of resources (49). Homoparental families, for their part, tackle discrimination and social stigma, factors that limit their economic opportunities and access to support networks, thus increasing their food vulnerability (50, 51). In contrast, nuclear and extended families tend to enjoy greater food security due to more equitable economic management and a more robust support system (52, 53). Although single-parent families are potentially vulnerable due to dependence on a single income and greater responsibilities, the literature has documented that many have developed strategies, such as the use of community support networks and the implementation of strict budgets, to mitigate the negative effects of FI (46, 54, 55). These strategies could explain why, in our study, no significant link was observed between single parenthood and FI, unlike what was observed in diverse families.

Our findings provide preliminary evidence on the potential influence of various family structures on food security, an area scarcely addressed in the current literature. Most previous studies focus on nuclear and single-parent households, leaving other family configurations unexamined in the context of FI. Our study employs a robust methodological approach, including a robust GLM adjusted to manage heteroscedasticity and outliers, which improves the reliability of the findings. In addition, the study is strengthened by a sample of 882 adolescents and utilization of validated tools, allowing generalization of findings within the context studied. Nevertheless, the study has limitations: the cross-sectional design precludes establishing causal relationships, and the use of self-reported data introduces potential recall and/or social desirability biases. On the other hand, geographic specificity limits the applicability of the results to other populations. Furthermore, although the model fits reasonably well (56), Nagelkerke’s *R*^2^ value (16.8%) indicates that the model explains only a small proportion of the variance, emphasizing the need for future studies to explore additional factors. Additionally, the Coronavirus Disease 2019 (COVID-19) pandemic may have influenced certain experiences, as data collection took place between 2021 and 2022. Despite these limitations, the study provides detailed insight into FI in adolescents from the *Valle de Ricote*, highlighting the importance of sociodemographic factors. Future longitudinal studies are recommended to understand the temporal dynamics of FI, establish the direction of the associations, and expand the variables investigated, to better understand the determinants of FI and develop effective interventions.

## Conclusion

The present findings demonstrate a high proportion of FI among adolescents highlighting the need for public policies aimed at improving access to nutritious and affordable food. FI was related to social and economic factors such as educational level, migration status, and family structure. Simplifying access to food assistance through multilingual resources, culturally appropriate foods, and cultural mediators is crucial for social inclusion. Additionally, promoting awareness campaigns and improving parental education through adult programs and nutrition literacy could enhance family nutrition management. Inclusive employment policies and reduced labor precarity may increase household income, while recognizing diverse family structures in social policies and strengthening community networks will help address FI. These findings enrich existing knowledge on FI in specific contexts and underscore the need for a comprehensive approach that addresses underlying vulnerabilities to develop effective, context-specific interventions.

## Data Availability

The original contributions presented in the study are included in the article/Supplementary material, further inquiries can be directed to the corresponding author.
