# Supplementary material for: Proportion of food insecurity and its sociodemographic correlates among Spanish adolescents: the EHDLA study
Source: Front Nutr. 2025 Mar 31;12:1527685. doi: 10.3389/fnut.2025.1527685 (PMC11994435; doi:10.3389/fnut.2025.1527685)
Supplement: Supplementary file 1 [file Table_1.docx]

## **Supplementary material**

## **Table S1**. Descriptive data of the study participants according to obesity status (N=882).

| **Variables** |  | **Very low food insecurity** | **Low food insecurity** | **Food secure** | ***p*-value ^†^** |
| --- | --- | --- | --- | --- | --- |
| Participants | *n* (%) |  |  |  |  |
| Age | Median (IQR) | 14.0 (2.0) | 14.0 (2.0) | 15.0 (2.0) | 0.011 |
| Sex | Boys | 323 (43.7) | 52 (44.4) | 14 (53.8) | 0.591 |
|  | Girls | 416 (56.3) | 65 (55.6) | 12 (46.2) |  |
| SES status | Low SES | 123 (16.6) | 44 (37.6) | 9 (34.6) | <0.001 |
|  | Medium SES | 405 (54.8) | 56 (47.9) | 11 (42.3) |  |
|  | High SES | 211 (28.6) | 17 (14.5) | 6 (23.1) |  |
| Immigrant status | Native | 599 (81.1) | 65 (55.6) | 11 (42.3) | <0.001 |
|  | Immigrant | 140 (18.9) | 52 (44.4) | 15 (57.7) |  |
| Type of schooling | Public | 535 (72.4) | 93 (79.5) | 18 (69.2) | 0.245 |
|  | Private with public funds | 204 (27.6) | 24 (20.5) | 8 (30.8) |  |
| Area of residence | Urban | 1.0 (1.0) | 1.0 (1.0) | 2.0 (1.8) | <0.001 |
|  | Rural | 3.0 (1.0) | 3.0 (1.0) | 4.0 (2.0) | 0.010 |
| Number of siblings | Median (IQR) | 645 (87.3) | 82 (70.1) | 15 (57.7) | <0.001 |
| Number of people at home | Median (IQR) | 94 (12.7) | 35 (29.9) | 11 (42.3) |  |
| Race/ethnicity | Caucasian | 205 (27.9) | 47 (40.5) | 12 (46.2) | 0.003 |
|  | Non-Caucasian | 301 (41.0) | 50 (43.1) | 9 (34.6) |  |
| Mother’s educational level | Primary education or lower | 228 (31.1) | 19 (16.4) | 5 (19.2) |  |
|  | Secondary education | 233 (32.6) | 56 (49.6) | 13 (56.5) | <0.001 |
|  | University education | 306 (42.8) | 45 (39.8) | 9 (39.1) |  |
| Father’s educational level | Primary education or lower | 176 (24.6) | 12 (10.6) | 1 (4.3) |  |
|  | Secondary education | 633 (85.7) | 80 (68.4) | 22 (84.6) | <0.001 |
|  | University education | 35 (4.7) | 9 (7.7) | 1 (3.8) |  |
| Type of family | Nuclear | 19 (2.6) | 6 (5.1) | 0 (0.0) |  |
|  | Single-parent | 52 (7.0) | 22 (18.8) | 3 (11.5) |  |
|  | Extended | 0.0 (0.0) | 3.0 (2.0) | 7.0 (1.0) | <0.001 |

CFSSM-S, Child Food Security Survey Module in Spanish; IQR, interquartile range; SES, socioeconomic status. ^†^ Statistical significance determined by Pearson chi-square (*χ*^2^) test (for categorical variables) or Kruskal-Wallis *H* test (for continuous variables).
